# The Presidency of the American Medical Association

**Published:** 1856-10

**Authors:** 


### EDITORIAL.

*The Presidency of the American Medical Association.*

Under this head we find the following as the leading editorial in the September number of the *Buffalo Medical and Surgical Journal*, viz:—

"It is evident that the time has arrived when some change in the manner of selecting the presiding officer of our national congress must be made. We have felt for years that some one should speak out upon the subject. We would have spoken a year ago, had not the fact that the meeting of the association, for 1856, was to be held at Detroit, and that the candidate then most prominent and since successful, had been in a position of personal antagonism with an interest which we represent, prevented us. Any attack made by us, at that time, would have been construed as personal, and as having its origin in local prejudices or jealousies; we therefore held our peace, and consented to forego our protest against an evil which must eventually impair the respectability of the association, and turn from it the regard of the best men in the profession.

"The next meeting is to be held at Nashville. We have none but friends in that locality, and we believe that they, too, will acquiesce in our plans for reform, or help to suggest another looking to the same end.

"The evil to which we allude is self-evident. Custom, not law, has made it the usual course to select the president of the association from the city in which the session is held. Thus the two meetings held at Philadelphia have given us Profs. Chapman and Wood, as presidents; that in St. Louis, Prof. Pope; that in Richmond, Dr. B. R. Wellford; and that in Detroit, Dr. Z. Pitcher. In only one instance has the rule failed to hold good. At the meeting in New York, in 1853, no one worthy of the office was found in that great city, and Dr. Jonathan Knight, of New Haven, was chosen. We hoped then that the precedent thus established in a large city, might be permitted to hold good in the small towns; but it is not so to be. It is evident, that until it is put out of the power of the nominating committee to virtually elect the president, we shall forever be subjected to the rule of that happy individual who happens to be the Nestor of the place of meeting. Grey hairs are honored at the expense of working men. Worse than this. To follow back the chain of promotion, we shall find that the association take the man the nominating committee choose to give them, and the committee take the man they are instructed to by the wire-pullers of the particular locality. In a small town, with from forty to fifty physicians, this matter can usually be cozily arranged among themselves, with more regard to their own interests than to the dignity of the association.

We regard the presidency of the American Medical Association as an honor too high to be lightly bestowed upon any man. The consideration of locality is the last that should be thought of. It is nonsense to make it a mere compliment to a town, in return for a good feed and abundant champagne. The occupant of that high station should be chosen from the profession at large. He who has labored; he who, through many years, has toiled to advance his profession; he who has built up for himself a name as a discoverer or medical philosopher; and who, perhaps, has found no other reward than a good name for all his efforts, such an one is our candidate.

"Under the present system, we can only elect him by accident. Such men as Bartlett, Drake, Morton, Parker, Mott, or Gross, can never be chosen under our present system. Those of them who are dead never would, and those who live never will, do the necessary work.

"The machinery by which presidents are made under the present constitution, is very simple. A special committee of one from each state is appointed by each delegation, to nominate all the officers. This special committee is a small body of men, and will never have the courage to break down the strong precedent now established. At the last meeting, Dr. Bloodgood, of Ill., offered a resolution, "That the constitution of this association be so amended as that hereafter the president shall be elected by ballot, and shall not be resident of the state in which he is elected."

"On motion of Dr. Watson, of N. Y., this was laid on the table. We do not like the resolution. The best man might be a resident of the state in which the meeting was held, and locality should not exclude him.

"What shall we do? The nominating committee is a bad piece of machinery in some respects, and a very good one in others. We have shown where its faults lie. It only requires a moment's thought to perceive that it must very much facilitate the selection of a place of meeting, and the nomination of committees. Indeed the argument of convenience is so strong, that we would willingly allow it the choice of all the officers except the presidency, reserving that as a distinguished honor to come direct from the association.

"We propose the following amendment to the constitution:—

"*Section IV. Officers.*

"The officers of the Association shall be a President, four Vice-Presidents, two Secretaries, and a Treasurer. *With the exception of the president*, they shall be nominated by a special committee of one member from each state represented at the meeting, and shall be elected by vote on a general ticket. *The president shall be chosen by ballot of all the members, the first ballot to be informal, and the person receiving the highest number on the second ballot, to be declared elected.*"

"Our amendment consists in the addition of the italicised portions to the present provisions. We throw it out to the profession at this time, in the hope that it may excite discussion in the Journals. We suppose that the motion of Dr. Watson, laying Dr. Bloodgood's resolution on the table, was superfluous; and that it may be properly brought up for discussion and amendment at the next meeting. Dr. B.'s resolution would have been laid on the table under the rule, had Dr. Watson withheld his motion. If we are right in this supposition, our proposition may come up at the Nashville meeting as an amendment to Dr. Bloodgood's.

"If we had any faith in committees, we should move simply to instruct them to be governed by the spirit of the constitution, which does not circumscribe their choice to any locality; but we have no such faith, and so resort to what seems to be the only practicable plan. As, under the present rule, the president is chosen early in the session, the proposed change can have no effect on next year's choice. We should, however, wish the amendment acted on before the selection of the place of meeting for 1858, for obvious reasons."

With the main sentiment of the above we fully concur. But it contains one or two errors which should be corrected.

It is not true that "in only *one* instance has the rule" of selecting the president from the place where the meeting was held, "failed to hold good." At the first regular anniversary meeting, held in the city of Baltimore, the President elected was Dr. Alexander H. Stevens, of New York. Neither was it true that at the meeting in New York in 1853, "no one was found in that great city *worthy* of the office," as asserted by our friend of the *Buffalo Journal.*

At that meeting in New York, the writer of this was a member of the nominating committee, and took an active part in the matter of selecting a candidate for President of the Association.

There were presented to the committee by the profession of New York, two candidates, namely, Dr. John W. Francis and Dr. Valentine Mott, both eminently *worthy* of being President of any Association. No member of the nominating committee objected to either of these men. Neither would there have been any objection to half a dozen other names that might have been selected from the profession in the "great city of New York." But several members of the committee did object strongly to the precedent, already established, of selecting a candidate necessarily from the place of meeting. Not only did members of the committee object to the propriety of the rule or precedent, but they claimed that then and there was the best possible *time* and *place* for repudiating it altogether. It was the best time, simply because the quicker a bad rule is broken the better. It was the best *place*, because the profession in New York having already furnished one President of the Association at a meeting in another city, would have less reason to complain on that account. The whole matter was fully and freely discussed in the committee, and when it recommended to the Association the election of Dr. Jonathan Knight, of New Haven, it was not from disrespect to New York or New Yorkers, but solely to repudiate a bad rule founded on precedent alone. We then hoped that subsequent committees would follow the example there set, and were much disappointed to find them refusing to do so at the next meeting held in St. Louis.

We are glad to see that the subject is at last attracting the attention of the profession.

For ourselves, we have heretofore uniformly opposed the practice of making the election of president depend on the accidental circumstance of location, and shall continue to do so, as long as we have any voice in the matter.

